# Maternal Antibiotic Treatment Disrupts the Intestinal Microbiota and Intestinal Development in Neonatal Mice

**DOI:** 10.3389/fmicb.2021.684233

**Published:** 2021-06-04

**Authors:** Chung-Ming Chen, Hsiu-Chu Chou, Yu-Chen S. H. Yang

**Affiliations:** ^1^Department of Pediatrics, Taipei Medical University Hospital, Taipei, Taiwan; ^2^Department of Pediatrics, School of Medicine, College of Medicine, Taipei Medical University, Taipei, Taiwan; ^3^Department of Anatomy and Cell Biology, School of Medicine, College of Medicine, Taipei Medical University, Taipei, Taiwan; ^4^Joint Biobank, Office of Human Research, Taipei Medical University, Taipei, Taiwan

**Keywords:** microbiota, intestine, tight junction, proliferating cell nuclear antigen, vascular endothelial growth factor

## Abstract

Maternal antibiotic treatment (MAT) during prenatal and intrapartum periods alters the bacterial composition and diversity of the intestinal microbiota of the offspring. The effect of MAT during pregnancy on the intestinal microbiota and its relationship with intestinal development remain unknown. This study investigated the effects of MAT during pregnancy on intestinal microbiota, injury and inflammation, vascularization, cellular proliferation, and the intestinal barrier in neonatal mice. At timed intervals, we fed pregnant C57BL/6N mice sterile drinking water containing antibiotics (ampicillin, gentamicin, and vancomycin; all 1 mg/ml) from gestational day 15 to delivery. The control dams were fed sterile drinking water. Antibiotic administration was halted immediately after birth. On postnatal day 7, the intestinal microbiota was sampled from the lower gastrointestinal tract and the ileum was harvested for histology, Western blot, and cytokines analyses. MAT significantly reduced the relative abundance of *Bacteroidetes* and *Firmicutes* and significantly increased the relative abundance of *Proteobacteria* in the intestine compared with their abundances in the control group. MAT also significantly increased intestinal injury score and cytokine levels, reduced the number of intestinal goblet cells and proliferating cell nuclear antigen-positive cells, and reduced the expressions of vascular endothelial growth factor and tight junction proteins. Therefore, we proposed that maternal antibiotic exposure during pregnancy disrupts the intestinal microbiota and intestinal development in neonatal mice.

## Introduction

Approximately 25% of women receive a course of antibiotics during pregnancy, and antibiotics account for 80% of medications used by pregnant women (Broe et al., 2014). They receive antibiotics to treat various conditions, such as bacterial vaginosis, urinary tract infections, and upper respiratory tract infections (Bookstaver et al., 2015). Although antibiotics are necessary for treating infections during pregnancy, they have short- and long-term effects. Maternal antibiotic exposure during pregnancy has been reported to be associated with lower rates of necrotizing enterocolitis and death in preterm infants and higher risks of asthma, atopic dermatitis, and hospitalization for infections in children (Blaser and Bello, 2014; Loewen et al., 2018; Miller et al., 2018; Reed et al., 2018; Alhasan et al., 2020).

The microbiota regulates inflammatory, infectious, and metabolic diseases and also causes, prevents, and sustains human diseases (Belkaid and Hand, 2014; Palm et al., 2015). Evidence suggests that host–microbe interactions may extend beyond the local environment to peripheral tissues (Marsland et al., 2015). Preclinical and human studies have demonstrated that maternal antibiotic administration during prenatal and intrapartum periods alters the bacterial composition and diversity of the offspring’s intestinal microbiota (Gonzalez-Perez et al., 2016; Yoshimoto et al., 2018; Dierikx et al., 2020; Zimmermann and Curtis, 2020). Microbiota profiles play a crucial role in intestinal barrier function and intestinal maturation in germ-free mice and human infants (Reinhardt et al., 2012; Sommer and Bäckhed, 2013; Kelly et al., 2015; Selma-Royo et al., 2020). However, the effects of maternal antibiotic treatment (MAT) during pregnancy on intestinal development and the relationship among MAT, the intestinal microbiota, and intestinal injury in neonates remain unknown. This study investigates the effects of MAT during pregnancy on intestinal microbiota and intestinal development in neonatal mice.

## Materials and Methods

### Animals and Experimental Protocol

Our study was approved by the Institutional Animal Care and Use Committee of Taipei Medical University (license number LAC-2019-0290) and according to an Association for Assessment and Accreditation of Laboratory Animal Care approved protocol. Six time-dated pregnant C57BL/6N mice were housed in individual metal cages with hardwood chip bedding on a 12-h light–dark cycle and free access to laboratory food of a standard chow diet (Rodent Laboratory Chow no. 5001, Ralston Purina Company, St. Louis, MO, United States) and water. The facility temperature was maintained at 20–23°C, and the relative humidity was between 36 and 57%; at the same time, minimal environmental stress and basic environmental enrichment were carried out in strict accordance with the recommendations with our institutional guidelines. The mice were allowed to deliver vaginally at term. At timed intervals, two mice in the MAT group were fed sterile drinking water containing antibiotics (ampicillin, gentamicin, and vancomycin; 1 mg/ml) often prescribed to pregnant women and human newborns, starting from gestational day 15 to delivery (Gray et al., 2017). Two control and two antibiotic-treated dams gave birth to 9 and 14 pups. The diet of the mothers was continued throughout the nursing time until the analysis of the offspring. Each mother was housed with her offspring in a single cage, and all neonates (both male and female) were breastfed throughout the study. The control dams were fed sterile drinking water. Antibiotic administration was halted immediately after birth. The current study was designed and reported in adherence to the ARRIVE Essential 10: Compliance Questionnaire.

### Mouse Tissue Collection

The animals were euthanized with an overdose of isoflurane on postnatal day 7. The abdomen was opened through a midline incision, 2 cm of the lower intestine from the anus to the colon was harvested, and the microbiota was sampled using a culture-independent approach (community sequencing of the 16S rRNA gene using the Illumina MiSeq). The instruments were rinsed with ethanol and flamed before each harvest, and the tissues were excised, placed in tubes containing 1 ml of sterile water, and homogenized mechanically using the Tissue-Tearor (BioSpec Products, Bartlesville, OK, United States). The tissue homogenizer was cleaned and rinsed with ethanol and water after each sample was obtained. Water control samples from homogenization that had been exposed to clean instruments were sequenced as procedural controls. The last 2 cm of the terminal ileum was excised, fixed with formalin, and embedded in paraffin for histological evaluation. A part of the ileum was flushed with saline to remove residual fecal matter and immediately fresh-frozen in liquid nitrogen for protein isolation.

### 16S rDNA Gene Sequencing and Next-Generation Sequencing Analysis

The protocol followed in this study for 16S rDNA analysis is described by Yang et al. (2020). In brief, 16S rDNA was purified from fecal samples with a QIAamp Fast DNA Stool Mini Kit (QIAGEN, Germany) and from lung tissues with a QIAamp DNA Microbiome Kit (QIAGEN, Germany). 16S rRNA gene amplification and library construction were performed according to the protocols provided by Illumina.^1^ V3-V4 region of bacterial 16S rRNA genes was amplified with the universal bacterial primers 341F (5'-CCTACGGGNGGCWGCAG-3') and 805R (5'-GACTACHVGG GTATCTAATCC-3') containing Illumina overhang adapter sequences in the forward (5'-TCGTCGGC AGCGTCAGATGTG TATAAGAGACAG-3') and reverse (5'-GTCTCGTGGGCT CGG AGAT GTGTATAAGAG ACAG-3') primers using a limited cycle PCR. Illumina sequencing adapters and dual-index barcodes were added to the amplicon target using a Nextera XT Index kit. Quantification and quality of the libraries were checked using a QSep100 Analyzer (BiOptic Inc., Taiwan). Finally, the libraries were normalized and pooled in an equimolar ratio and sequenced with an Illumina MiSeq sequencer. The code of the 16S analysis was supported by Dr. IH Lin, and the information could be found at the Web site https://github.com/ycl6/16S-rDNA-V3-V4. The gene-specific sequences used in this protocol target the 16S V3 and V4 regions and were removed from the demultiplexed, paired reads using Cutadapt (v 1.12). The filtered reads were processed in the R environment (v 3.6.1) using R package DADA2 (v 1.14.1) following the procedure described by Callahan et al. (2016). Briefly, the forward and reversed reads were filtered and trimmed based on the read quality score and read length. Dereplication was then performed to merge identical reads; then, reads were subjected to the denoise DADA2 algorithm which alternate between error-rate estimation and sample composition inference until they converge on a jointly consistent solution. Finally, the paired reads were merged that required a minimal of 20 bp overlap and chimeras were subsequently removed. Taxonomy assignment was performed using the SILVA database (v 138) as a reference, with minimum bootstrap confidence of 80. Multiple sequence alignment of the SVs was performed using DECIPHER (v 2.14.0), and a phylogenetic tree was constructed from the alignment using phangorn, v 2.5.5 (Schliep, 2011; Quast et al., 2013). The count table, taxonomy assignment results, and phylogenetic tree were consolidated into a phyloseq object, community analysis, and Bray–Curtis distances were performed using phyloseq (v 1.30.0; McMurdie and Holmes, 2013). The alpha diversity indices were calculated using the estimate_richness function of the phyloseq package. The adonis and betadisper functions from the vegan package (v 2.5.6) were used for statistical analysis of the dissimilarity of composition between groups and homogeneity of dispersion, respectively. The enrichment analysis between groups was analysed using the linear discriminant analysis (LDA) effect size (LEfSe) method with Wilcoxon-Mann-Whitney test (at α = 0.05) and logarithmic LDA score more than 2 (Segata et al., 2011). Sequence reads were input into the European Nucleotide Archive under the accession number PRJEB43913.

### Histology

The ileum was separated into 5-μm sections, stained with hematoxylin and eosin, and examined through light microscopy for evaluation of intestinal morphology. The intestinal mucosal injury was scored on a scale of 0–5, where 0 = normal mucosal villi, 1 = subepithelial space at the villus tips and frequent capillary congestion, 2 = extension of the subepithelial space with the moderate lifting of the epithelial layer from the lamina propria, 3 = massive epithelial lifting down the sides of the villi with occasionally denuded villi tips, 4 = denuded villi with dilation of the lamina propria and capillaries, and 5 = disintegration of the lamina propria, hemorrhage, and ulceration (Chiu et al., 1970). The sections for goblet cell quantification were stained with Alcian blue staining, and the number of goblet cells per 100 epithelial cells was counted (Elgin et al., 2019).

### Immunohistochemistry

After routine deparaffinization, the slides were immersed in a 0.01 mol/L sodium citrate buffer (pH 6.0) for heat-induced epitope retrieval. To block endogenous peroxidase activity and nonspecific antibody binding, the sections were preincubated for 1 h at room temperature in 0.1 mol/L phosphate-buffered saline containing 10% normal goat serum and 0.3% H_2_O_2_. All sections were incubated with the following primary antibodies for 20 h at 4°C: mouse monoclonal antioccludin, rat monoclonal anti-ZO-1 (both antioccludin and anti-ZO-1 were 1:20 diluted; Santa Cruz Biotechnology Inc., CA, United States), rabbit monoclonal anti-proliferating cell nuclear antigen (anti-PCNA; 1:100; Abcam, Cambridge, MA, United States), mouse monoclonal anti-vascular endothelial growth factor (anti-VEGF) and anti-CD68, and rat monoclonal anti-CD45R (all three antibodies were diluted at a 1:50 ratio; Santa Cruz Biotechnology). The sections were then treated for 1 h at 37°C with biotinylated goat anti-mouse IgG, goat anti-rat IgG, or goat anti-rabbit IgG (1:200; Jackson ImmunoResearch Laboratories Inc., PA, United States) before undergoing a reaction with reagents from an avidin–biotin complex kit (Vector, CA, United States). A diaminobenzidine substrate kit (Vector, CA, United States) was used to visualize the brown reaction products in accordance with the manufacturer’s instructions. All immunostained sections were viewed and photographed using an Olympus BX43 microscope. The quantification method for PCNA-positive cells and CD45- and CD68-positive stained cells was modified from Şensoy and Öznurlu (2019), the cells were counted in a total of 100 crypt cells, and the results were recorded as the percentage (%) of positive cells.

### Intestinal Cytokine Levels

Intestinal tissues were homogenized in 1 ml of ice-cold lysis buffer containing 1% Nonidet P-40, 0.1% sodium dodecyl sulfate, 0.01 m deoxycholic acid, and a complete protease cocktail inhibitor. Cell extracts were centrifuged, and the levels of interleukin (IL)-1β and IL-6 in supernatants were measured with an enzyme-linked immunosorbent assay kit (Cloud-Clone Corp., Houston, TX, United States).

### Statistical Analysis

All data are presented as the mean ± SD. A test for normality of variances was conducted using the Shapiro–Wilk test. Normally distributed data were analyzed using Student’s *t*-test. Non-normally distributed data were analyzed using the Mann–Whitney *U* test. The Kaplan–Meier method was used to determine the survival rate, and a log-rank test was used to compare groups. Differences were considered statistically significant at *p* < 0.05.

## Results

### Total Intestinal Commensal Bacteria at Birth, and Survival Rate and Body Weight on Postnatal Day 7

**Figure 1:**
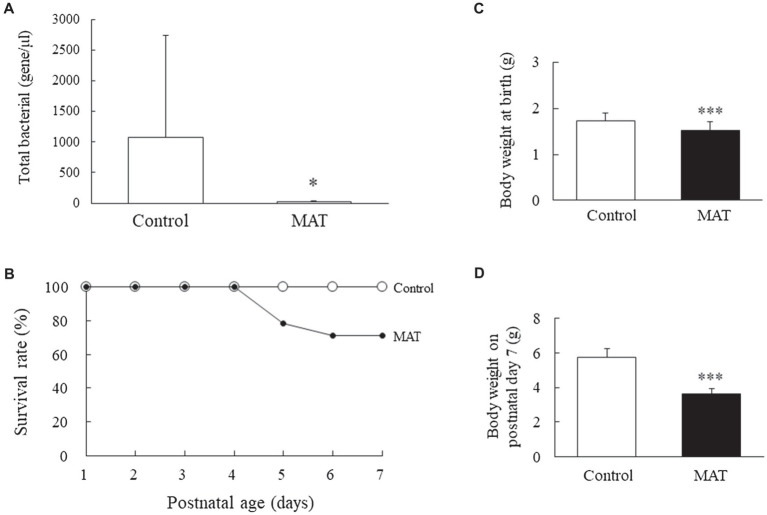
Effects of MAT on **(A)** total intestinal commensal bacteria at birth, **(B)** survival rate, **(C)** body weight at birth, and **(D)** body weight on postnatal day 7. MAT significantly reduced the number of commensal bacteria in newborn mice compared with that in the control newborns. The survival rates were comparable between the mice born to control and antibiotic-treated dams. The mice born to the antibiotic-treated dams exhibited significantly lower body weights at birth and on postnatal day 7 than that of the mice born to the control dams. ^*^p < 0.05; ^***^p < 0.001. MAT, maternal antibiotic treatment.

Maternal antibiotic treatment significantly reduced the number of commensal bacteria in newborn mice compared with control newborns (*p* < 0.05; Figure 1A). Three control and three antibiotic-treated dams gave birth to 16 and 19 pups, respectively. At birth, seven mice from the control group and five mice from the MAT group were killed and their lower intestinal tract was used to determine the total bacterial load, which was quantified using qPCR with universal bacterial primers (forward: 5'-AAACTCAAAKGAATTGACGG-3'; reverse: 5'-CTCACRRCACGAGCTGA-3'). The rest nine mice born to the control dams survived (Figure 1B). Three mice and one mouse born to the antibiotic-treated dams died, on postnatal days 5 and 6, respectively. The mice born to control and antibiotic-treated dams had comparable survival rates. The mice born to the antibiotic-treated dams had significantly lower body weights at birth and on postnatal day 7 than did the mice born to the control dams (*p* < 0.001; Figures 1C,D).

### MAT Altered the Intestinal Microbiota Composition

**Figure 2:**
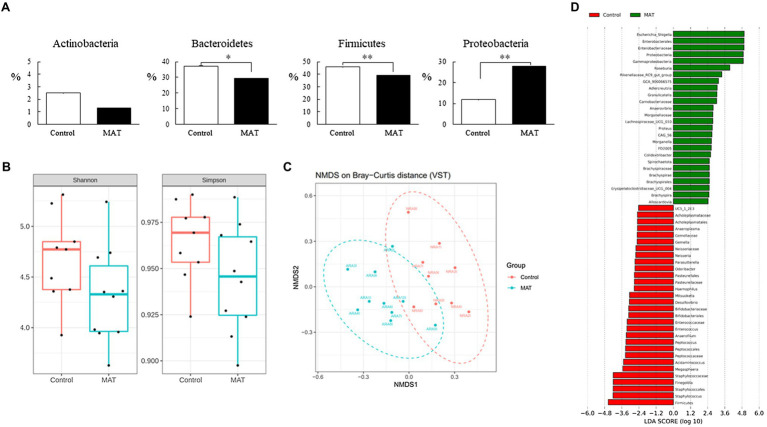
Effects of MAT on **(A)** bacterial composition at the phylum level, **(B)** α diversity, **(C)** NMDS, and **(D)** histogram of the linear discriminant analysis scores of bacterial at the family level of the intestinal microbiota in 7-day-old mice. MAT altered the bacterial composition and diversity of the intestinal microbiota in 7-day-old offspring. MAT significantly reduced the relative abundances of *Bacteroidetes* and *Firmicutes* and significantly increased the relative abundance of *Proteobacteria* in the intestine compared with the abundances in the control group. No significant differences in Shannon diversity or Simpson diversity were observed between the control and MAT groups. NMDS revealed that the intestinal microbiome in the control group was significantly different from that in the MAT group. Bacterial taxa significantly differed across the control and MAT groups identified by LEfSe using the default parameters. ^*^p < 0.05; ^**^p < 0.01. NMDS, nonmetric multidimensional scaling; and MAT, maternal antibiotic treatment.

We analyzed the taxonomic community structure of the intestinal microbiome on postnatal day 7 to determine its response to MAT (Figure 2A). At the phylum level, the intestinal microbiome in the control and MAT groups contained four major bacterial phyla (%): *Actinobacteria* (2.5 ± 3.1 and 1.3 ± 1.3, respectively), *Bacteroidetes* (37.3 ± 8.2 and 29.2 ± 8.3, respectively), *Firmicutes* (45.9 ± 4.7 and 39.2 ± 3.0, respectively), and *Proteobacteria* (11.8 ± 7.5 and 27.8 ± 10.8, respectively). The first three phyla accounted for >95% of the sequences in the two groups. MAT significantly reduced the relative abundance of *Bacteroidetes* (*p* < 0.05) and *Firmicutes* (*p* < 0.01) and significantly increased the relative abundance of *Proteobacteria* (*p* < 0.01) in the intestine compared with the control group. Although the intestine microbiota composition was distinct between the control and MAT groups, MAT group exhibited lower Shannon diversity and Simpson diversity and the differences were not statistically significant (*p* = 0.079 and *p* = 0.11, respectively; Figure 2B). Nonmetric multidimensional scaling revealed that the intestinal microbiome in the control group was significantly different from that in the MAT group (*p* < 0.000999; Figure 2C). To identify microbial taxa affected by MAT, linear discriminant analysis effect size (LEfSe) analysis was performed, which revealed significant differences in relative bacterial abundance at the family level (Figure 2D).

### MAT Induced the Intestinal Injury and Reduced Tight Junction Protein Expression

**Figure 3:**
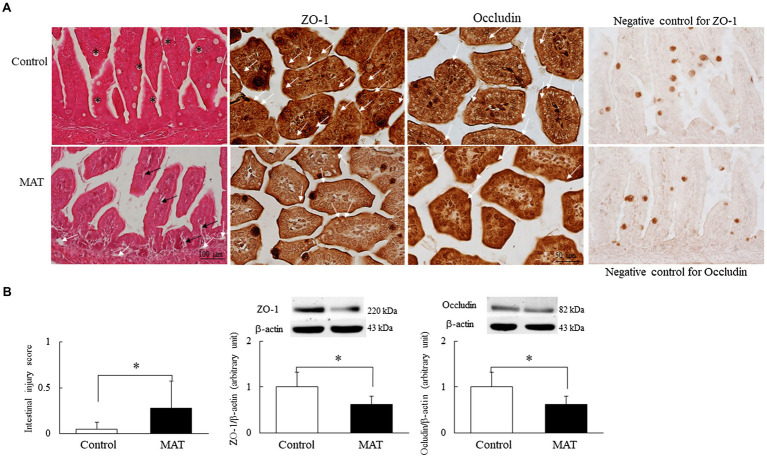
Effects of MAT on **(A)** immunohistochemistry of the ileum and **(B)** intestinal injury score and Western blotting of tight junction proteins in 7-day-old mice. Compared with the mice born to the control dams, the mice born to the antibiotic-treated dams exhibited fewer goblet cells (asterisk) in the epithelium and a significantly higher intestinal injury score; they also exhibited dilated capillaries and lacteal ducts in the lamina propria of the villi (black arrow) and separated lamina propria in the basal portion of the mucosa from the submucosa (white arrow). Mice born to the control dams exhibited intact construct of ZO-1 and occludin staining (white arrow). Those born to the antibiotic-treated dams exhibited disrupted ZO-1 and occludin immunohistochemistry between adjacent enterocytes and significantly lower ZO-1 and occludin protein expression levels than the mice born to the control dams (*n* = 9). ^*^p < 0.05. MAT, maternal antibiotic treatment.

Representative immunohistochemistry and Western blotting results for occludin and ZO-1 are presented in Figure 3. The mice born to the control dams exhibited a normal intestinal wall configuration, a well-defined striated border at the luminal surface, and prominent goblet cells in the epithelium (Figure 3A). Those born to antibiotic-treated dams exhibited fewer goblet cells in the epithelium, dilated capillaries and lacteal ducts in the lamina propria of the villi, separated lamina propria in the basal portion of the mucosa from the submucosa, and a significantly (5.5-fold) higher intestinal injury score compared with the mice born to the control dams (*p* < 0.05; Figure 3B). The immunoreactivity of ZO-1 and occludin was observed on the cell membrane between adjacent intestinal epithelial cells (Figure 3A). We observed an intact construct of ZO-1 and occludin staining in the mice born to the control dams. The mice born to the antibiotic-treated dams exhibited disrupted ZO-1 and occludin immunohistochemistry between adjacent enterocytes and significantly (0.4-fold) lower ZO-1 and occludin protein expression levels compared with the mice born to the control dams (*p* < 0.05, Figure 3B).

### MAT Reduced the Numbers of Intestinal Goblet Cells and PCNA-Positive Cells and VEGF Protein Expression

**Figure 4:**
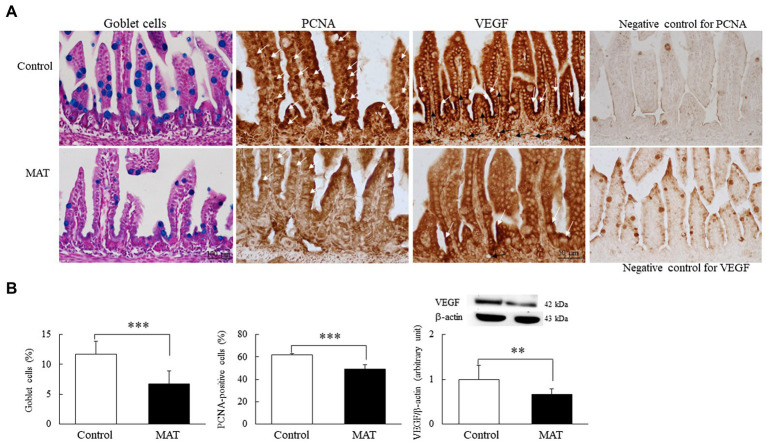
Effects of MAT on **(A)** immunohistochemistry results and **(B)** results of quantitative analysis of goblet cells and PCNA-positive cells and Western blotting of VEGF in 7-day-old mice. The immunoreactivity for PCNA (white arrow) was localized to the nucleus and appeared along the epithelium of the intestinal mucosa and in the basal area of the intestinal crypts. Dark brown VEGF-positive staining (white arrow) was localized at the apical cytoplasm of the epithelial cells in both groups, and the VEGF-positive endothelial cells (black arrow) of blood vessels were observed in the lamina propria of the control group. The mice born to the control dams demonstrated more prominent immunoreactivity for PCNA and VEGF staining. The mice born to the antibiotic-treated dams exhibited significantly fewer mucus-positive goblet cells (blue stained) and PCNA-positive cells and significantly lower VEGF protein expression than did the mice born to the control dams (*n* = 9). ^**^p < 0.01; ^***^p < 0.001. MAT, maternal antibiotic treatment; PCNA, proliferating cell nuclear antigen; and VEGF, vascular endothelial growth factor.

We quantified the goblet cells and PCNA-positive cells to assess the components of innate immunity and proliferation in the intestine, respectively (Figure 4A). The immunoreactivity for PCNA was localized to the nucleus and was observed along the epithelium of the intestinal mucosa and in the basal area of the intestinal crypts. Dark brown VEGF-positive staining was localized at the apical cytoplasm of the epithelial cells in both groups; only mice born to the control dams displayed positive results for the immunostaining of VEGF on the endothelial cells of blood vessels in the lamina propria. The mice born to the control dams exhibited more prominent immunoreactivity for PCNA and VEGF staining. Those born to the antibiotic-treated dams had significantly (0.7-fold, *p* < 0.001) fewer mucus-positive goblet cells and PCNA-positive cells and significantly (0.66-fold, *p* < 0.01) lower VEGF protein expression than did the mice born to the control dams (Figure 4B).

### MAT Increased the Numbers of Intestinal CD45- and CD68-Positive Cells and Cytokines

**Figure 5:**
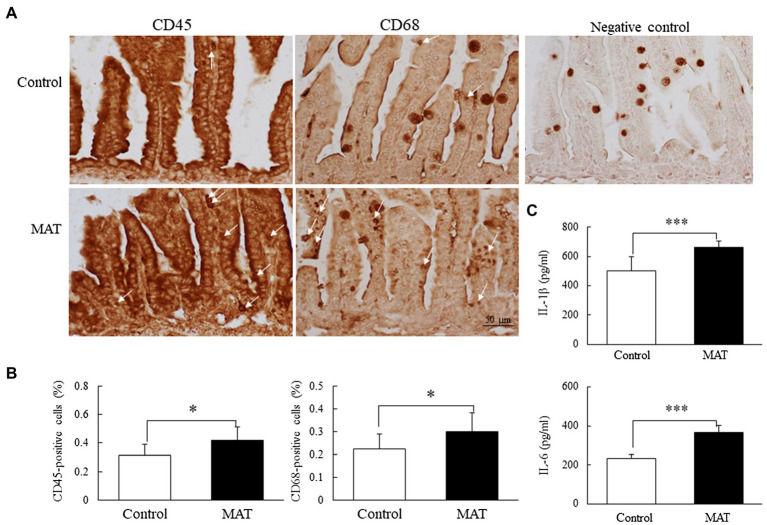
Effects of MAT on **(A)** immunohistochemistry results, **(B)** results of quantitative analysis of CD45 and CD68 cells, and **(C)** cytokines in 7-day-old mice. CD45 and CD68 immunoreactivity was observed on the cytoplasm and cell membrane of the small lymphocytes. The CD45- and CD68-positive cells (white arrow) were scattered over the lamina propria and between the epithelial cells of the intestinal mucosa. The mice born to antibiotic-treated dams exhibited significantly higher percentages of CD45- and CD68-positive cells and higher IL-1β and IL-6 expression levels than did those born to control dams. ^*^p < 0.05; ^***^p < 0.001. MAT, maternal antibiotic treatment.

CD45 and CD68 immunoreactivity was observed on the cytoplasm and the cell membrane of the small lymphocytes (Figure 5A). The CD45- and CD68-positive cells were scattered over the lamina propria of the villi and between the epithelial cells of the intestinal mucosa. The mice born to the antibiotic-treated dams exhibited a significantly (1.35-fold, *p* < 0.05) higher percentage of CD45- and CD68-positive cells and (1.32-fold and 1.57-fold, respectively, *p* < 0.001) higher IL-1β and IL-6 expression levels than those born to the control dams (Figures 5B,C).

## Discussion

Our *in vivo* model demonstrated that MAT during pregnancy suppressed the intestinal microbiota and reduced the body weight of offspring at birth. Maternal antibiotic exposure altered the bacterial composition of intestinal microbiota, induced intestinal injury and inflammation, inhibited vascularization and cellular proliferation, and disrupted the intestinal barrier in neonatal mice. These findings suggest that the administration of antibiotics to women during pregnancy and the subsequent alterations to intestinal microbiota and intestinal development can cause short-term adverse effects.

Rat pups born to dams treated with perinatal antibiotics (amoxicillin or vancomycin) from day 8 prior to delivery to postnatal week 4 gained less weight than did pups born to control dams (Tulstrup et al., 2018). However, the effects of maternal antibiotic exposure during pregnancy on postnatal body weights are unknown. In this study, mice exposed to prenatal maternal antibiotics (ampicillin, gentamicin, and vancomycin) from gestational day 15 to delivery exhibited significantly lower body weights on postnatal day 7 than did mice exposed to sterile drinking water. This effect was not dependent on litter size but was associated with a reduced number of commensal bacteria in newborn mice born to antibiotic-treated dams. These results suggest that maternal antibiotic exposure during pregnancy alters body growth in the postnatal period, indicating that in utero antibiotic exposure alters organogenesis (Vidal et al., 2013). We demonstrated that MAT exposure increases mice mortality, although the survival rates were comparable between the mice born to the control dams and those born to the antibiotic-treated dams.

In this study, we found that MAT during pregnancy suppressed the development of the intestinal microbiota and disrupted intestinal development, as demonstrated by decreased numbers of goblet cells and PCNA-positive cells and decreased VEGF and tight junction protein expression. These results support the previous findings that the intestinal microbiota is crucial for intestinal development through the promotion of mucus production, cellular proliferation, vascularization, and preservation of epithelial junctions in germ-free mice and human infants (Reinhardt et al., 2012; Sommer and Bäckhed, 2013; Kelly et al., 2015; Selma-Royo et al., 2020). These findings suggest that antibiotics administration during pregnancy should be cautious and the manipulation of the intestinal microbiota might potentially prevent intestinal injury and promote intestinal development in antibiotic-exposed infants.

We demonstrated that MAT during pregnancy increases intestinal cytokine (IL-1β and IL-6) levels in neonatal mice. We also quantified intestinal lymphocyte populations of CD45- and CD68-positive stained cells and demonstrated that MAT during pregnancy increases the numbers of these cells. The results suggest that CD45 and CD68 cells produce cytokines, which is compatible with the finding that type 3 innate lymphoid cells in the neonatal intestine produce the cytokines IL-17 and IL-22 (Niu et al., 2020).

In this study, we used a combination of ampicillin, gentamicin, and vancomycin because they are the most commonly prescribed antibiotics during pregnancy (Mylonas, 2011). We demonstrated that MAT during pregnancy altered the intestinal bacterial composition, induced intestinal injury and inflammation, and disrupted the intestinal development in neonatal mice. These findings suggest that antibiotics should only be applied to pregnant women if antibiotics are really needed. Further studies are needed to evaluate the effect of each antibiotic on intestinal microbiota and development in the offspring.

Our study has several limitations. First, we did not evaluate the maternal intestinal microbiota at the end of antibiotic treatment. Studies have demonstrated that maternal antibiotic exposure during pregnancy disturbs the maternal and fetal intestinal microbiota and affects the health of the offspring (Calatayud et al., 2019; Younge et al., 2019). However, the effects of antibiotics on intestine development in pregnant mice were largely unknown. Second, we did not evaluate the effects of MAT during pregnancy on intestinal permeability in the offspring, though studies have demonstrated that MAT increases colonic permeability on postnatal day 14 in swine offspring and that a disrupted intestinal barrier is associated with impaired intestinal function in hyperoxia-exposed newborn rats (Arnal et al., 2015; Chou and Chen, 2017). Third, maternal antibiotic administration was halted immediately after birth and we measured the intestinal microbiota and intestinal development on postnatal day 7. We did not investigate the effects of MAT during pregnancy through immediate postnatal period in the offspring mice. These might explain the differences in bacterial composition, and tight junction protein expression between the control and MAT groups was not large. Fourth, we did not measure immunological parameters to elucidate the mechanisms underlying impaired intestinal development.

## Conclusion

In conclusion, this study demonstrated that MAT during pregnancy alters the intestinal microbiota, increases intestinal injury and inflammation, inhibits intestinal cellular proliferation and vascularization, and disrupts the intestinal barrier in neonatal mice. Research on intestinal development after prenatal antibiotic exposure in neonatal mice will increase our understanding of intestinal barrier function and provide strategies for the prevention of long-term adverse effects of MAT during pregnancy. Avoiding antibiotics during pregnancy and the manipulation of intestinal microbiota has the potential to prevent and treat the intestinal injury in infants exposed to prenatal antibiotics. Further studies are required to explore the mechanisms that connect MAT during pregnancy with impaired intestinal development in neonates.

## Data Availability Statement

The datasets presented in this study can be found in online repositories. The names of the repository and accession number(s) can be found at European Nucleotide Archive under the accession number PRJEB43913.

## Ethics Statement

This animal study was approved by the Institutional Animal Care and Use Committee of Taipei Medical University (LAC-2019-0290).

## Author Contributions

C-MC: study design, acquisition and analysis of the data, and drafting and final approval of the manuscript. H-CC and Y-CY: acquisition and analysis of the data, and drafting and final approval of the manuscript. All authors contributed to the article and approved the submitted version.

### Conflict of Interest

The authors declare that the research was conducted in the absence of any commercial or financial relationships that could be construed as a potential conflict of interest.

## Footnotes

^1^https://support.illumina.com/downloads/16S_metagenomic_sequencing_library_preparation.html

## References

[ref1] Alhasan M. M. Cait A. M. Heimesaat M. M. Blaut M. Klopfleisch R. Wedel A.. (2020). Antibiotic use during pregnancy increases offspring asthma severity in a dose-dependent manner. Allergy 75, 1979–1990. 10.1111/all.14234, PMID: 32064643

[ref2] Arnal M. E. Zhang J. Erridge C. Smidt H. Lallès J. P. (2015). Maternal antibiotic-induced early changes in microbial colonization selectively modulate colonic permeability and inducible heat shock proteins, and digesta concentrations of alkaline phosphatase and TLR-stimulants in swine offspring. PLoS One 10:e0118092. 10.1371/journal.pone.0118092, PMID: 25689154 PMC4331088

[ref4] Belkaid Y. Hand T. W. (2014). Role of the microbiota in immunity and inflammation. Cell 157, 121–141. 10.1016/j.cell.2014.03.011, PMID: 24679531 PMC4056765

[ref5] Blaser M. J. Bello M. G. (2014). Maternal antibiotic use and risk of asthma in offspring. Lancet Respir. Med. 2:e16. 10.1016/S2213-2600(14)70219-X, PMID: 25298059

[ref6] Bookstaver P. B. Bland C. M. Griffin B. Stover K. R. Eiland L. S. McLaughlin M.. (2015). A review of antibiotic use in pregnancy. Pharmacotherapy 35, 1052–1062. 10.1002/phar.1649, PMID: 26598097

[ref7] Broe A. Pottegård A. Lamont R. F. Jørgensen J. S. Damkier P. (2014). Increasing use of antibiotics in pregnancy during the period 2000–2010: prevalence, timing, category, and demographics. BJOG 121, 988–996. 10.1111/1471-0528.12806, PMID: 24754708

[ref8] Calatayud M. Koren O. Collado M. C. (2019). Maternal microbiome and metabolic health program microbiome development and health of the offspring. Trends Endocrinol. Metab. 30, 735–744. 10.1016/j.tem.2019.07.021, PMID: 31493988

[ref9] Callahan B. J. Sankaran K. Fukuyama J. A. McMurdie P. J. Holmes S. P. (2016). Bioconductor workflow for microbiome data analysis: from raw reads to community analyses. F1000Res. 5:1492. 10.12688/f1000research.8986.1, PMID: 27508062 PMC4955027

[ref11] Chiu C. J. Scott H. J. Gurd F. N. (1970). Intestinal mucosal lesion in low-flow states. II. The protective effect of intraluminal glucose as energy substrate. Arch. Surg. 101, 484–488. 10.1001/archsurg.1970.01340280036010, PMID: 5311679

[ref12] Chou H. C. Chen C. M. (2017). Neonatal hyperoxia disrupts the intestinal barrier and impairs intestinal function in rats. Exp. Mol. Pathol. 102, 415–421. 10.1016/j.yexmp.2017.05.006, PMID: 28506763

[ref13] Dierikx T. H. Visser D. H. Benninga M. A. van Kaam A. H. L. C. de Boer N. K. H. de Vries R.. (2020). The influence of prenatal and intrapartum antibiotics on intestinal microbiota colonisation in infants: a systematic review. J. Infect. 81, 190–204. 10.1016/j.jinf.2020.05.002, PMID: 32389786

[ref14] Elgin T. G. Fricke E. M. Gong H. Reese J. Mills D. A. Kalantera K. M.. (2019). Fetal exposure to maternal inflammation interrupts murine intestinal development and increases susceptibility to neonatal intestinal injury. Dis. Model. Mech. 12:dmm040808. 10.1242/dmm.040808, PMID: 31537532 PMC6826024

[ref15] Gonzalez-Perez G. Hicks A. L. Tekieli T. M. Radens C. M. Williams B. L. Lamousé-Smith E. S.. (2016). Maternal antibiotic treatment impacts development of the neonatal intestinal microbiome and antiviral immunity. J. Immunol. 196, 3768–3779. 10.4049/jimmunol.1502322, PMID: 27036912

[ref16] Gray J. Oehrle K. Worthen G. Alenghat T. Whitsett J. Deshmukh H.. (2017). Intestinal commensal bacteria mediate lung mucosal immunity and promote resistance of newborn mice to infection. Sci. Transl. Med. 9:eaaf9412. 10.1126/scitranslmed.aaf9412, PMID: 28179507 PMC5880204

[ref17] Kelly C. J. Zheng L. Campbell E. L. Saeedi B. Scholz C. C. Bayless A. J.. (2015). Crosstalk between microbiota-derived short-chain fatty acids and intestinal epithelial HIF augments tissue barrier function. Cell Host Microbe 17, 662–671. 10.1016/j.chom.2015.03.005, PMID: 25865369 PMC4433427

[ref18] Loewen K. Monchka B. Mahmud S. M. Azad M. B. (2018). Prenatal antibiotic exposure and childhood asthma: a population-based study. Eur. Respir. J. 52:1702070. 10.1183/13993003.02070-2017, PMID: 29678946

[ref19] Marsland B. J. Trompette A. Gollwitzer E. S. (2015). The gut-lung axis in respiratory disease. Ann. Am. Thorac. Soc. 12(Suppl. 2), S150–S156. 10.1513/AnnalsATS.201503-133AW 26595731

[ref20] McMurdie P. J. Holmes S. (2013). Phyloseq: an R package for reproducible interactive analysis and graphics of microbiome census data. PLoS One 8:e61217. 10.1371/journal.pone.0061217, PMID: 23630581 PMC3632530

[ref21] Miller J. E. Wu C. Pedersen L. H. de Klerk N. Olsen J. Burgner D. P.. (2018). Maternal antibiotic exposure during pregnancy and hospitalization with infection in offspring: a population-based cohort study. Int. J. Epidemiol. 47, 561–571. 10.1093/ije/dyx272, PMID: 29415232

[ref22] Mylonas I. (2011). Antibiotic chemotherapy during pregnancy and lactation period: aspects for consideration. Arch. Gynecol. Obstet. 283, 7–18. 10.1007/s00404-010-1646-3, PMID: 20814687

[ref23] Niu X. Daniel S. Kumar D. Ding E. Y. Savani R. C. Koh A. Y.. (2020). Transient neonatal antibiotic exposure increases susceptibility to late-onset sepsis driven by microbiota-dependent suppression of type 3 innate lymphoid cells. Sci. Rep. 10:12974. 10.1038/s41598-020-69797-z 32737397 PMC7395748

[ref24] Palm N. W. de Zoete M. R. Flavell R. A. (2015). Immune-microbiota interactions in health and disease. Clin. Immunol. 159, 122–127. 10.1016/j.clim.2015.05.014, PMID: 26141651 PMC4943041

[ref25] Quast C. Pruesse E. Yilmaz P. Gerken J. Schweer T. Yarza P.. (2013). The SILVA ribosomal RNA gene database project: improved data processing and web-based tools. Nucleic Acids Res. 41, D590–D596. 10.1093/nar/gks1219, PMID: 23193283 PMC3531112

[ref26] Reed B. D. Schibler K. R. Deshmukh H. Ambalavanan N. Morrow A. L. (2018). The impact of maternal antibiotics on neonatal disease. J. Pediatr. 197, 97–103. 10.1016/j.jpeds.2018.01.056, PMID: 29551319 PMC6028045

[ref27] Reinhardt C. Bergentall M. Greiner T. U. Schaffner F. Ostergren-Lundén G. Petersen L. C.. (2012). Tissue factor and PAR1 promote microbiota-induced intestinal vascular remodelling. Nature 483, 627–631. 10.1038/nature10893, PMID: 22407318 PMC3885420

[ref28] Schliep K. P. (2011). Phangorn: phylogenetic analysis in R. Bioinformatics 27, 592–593. 10.1093/bioinformatics/btq706, PMID: 21169378 PMC3035803

[ref29] Segata N. Izard J. Waldron L. Gevers D. Miropolsky L. Garrett W. S.. (2011). Metagenomic biomarker discovery and explanation. Genome Biol. 12:R60. 10.1186/gb-2011-12-6-r60 21702898 PMC3218848

[ref30] Selma-Royo M. Calatayud Arroyo M. García-Mantrana I. Parra-Llorca A. Escuriet R. Martínez-Costa C.. (2020). Perinatal environment shapes microbiota colonization and infant growth: impact on host response and intestinal function. Microbiome 8:167. 10.1186/s40168-020-00940-8 33228771 PMC7685601

[ref31] Şensoy E. Öznurlu Y. (2019). Determination of the changes on the small intestine of pregnant mice by histological, enzyme histochemical, and immunohistochemical methods. Turk. J. Gastroenterol. 30, 917–924. 10.5152/tjg.2019.18681, PMID: 31625934 PMC6812947

[ref32] Sommer F. Bäckhed F. (2013). The gut microbiota-masters of host development and physiology. Nat. Rev. Microbiol. 11, 227–238. 10.1038/nrmicro2974, PMID: 23435359

[ref33] Tulstrup M. V. Roager H. M. Thaarup I. C. Frandsen H. L. Frøkiær H. Licht T. R.. (2018). Antibiotic treatment of rat dams affects bacterial colonization and causes decreased weight gain in pups. Commun. Biol. 1:145. 10.1038/s42003-018-0140-5 30272021 PMC6137057

[ref34] Vidal A. C. Murphy S. K. Murtha A. P. Schildkraut J. M. Soubry A. Huang Z.. (2013). Associations between antibiotic exposure during pregnancy, birth weight and aberrant methylation at imprinted genes among offspring. Int. J. Obes. 37, 907–913. 10.1038/ijo.2013.47, PMID: 23609933 PMC3705584

[ref35] Yang Y. S. H. Chang H. W. Lin I. H. Chien L. N. Wu M. J. Liu Y. R.. (2020). Long-term proton pump inhibitor administration caused physiological and microbiota changes in rats. Sci. Rep. 10:866. 10.1038/s41598-020-57612-8, PMID: 31964941 PMC6972906

[ref36] Yoshimoto A. Uebanso T. Nakahashi M. Shimohata T. Mawatari K. Takahashi A.. (2018). Effect of prenatal administration of low dose antibiotics on gut microbiota and body fat composition of newborn mice. J. Clin. Biochem. Nutr. 62, 155–160. 10.3164/jcbn.17-53, PMID: 29610555 PMC5874232

[ref37] Younge N. McCann J. R. Ballard J. Plunkett C. Akhtar S. Araújo-Pérez F.. (2019). Fetal exposure to the maternal microbiota in humans and mice. JCI Insight 4:e127806. 10.1172/jci.insight.127806, PMID: 31479427 PMC6795398

[ref38] Zimmermann P. Curtis N. (2020). Effect of intrapartum antibiotics on the intestinal microbiota of infants: a systematic review. Arch. Dis. Child. Fetal Neonatal Ed. 105, 201–208. 10.1136/archdischild-2018-316659, PMID: 31296695

